# *Direct Repeat 6* from Human Herpesvirus-6B Encodes a Nuclear Protein that Forms a Complex with the Viral DNA Processivity Factor p41

**DOI:** 10.1371/journal.pone.0007457

**Published:** 2009-10-15

**Authors:** Mariane H. Schleimann, Janni M. L. Møller, Emil Kofod-Olsen, Per Höllsberg

**Affiliations:** Department of Medical Microbiology and Immunology, Aarhus University, Aarhus, Denmark; University of Minnesota, United States of America

## Abstract

The *SalI*-L fragment from human herpesvirus 6A (HHV-6A) encodes a protein DR7 that has been reported to produce fibrosarcomas when injected into nude mice, to transform NIH3T3 cells, and to interact with and inhibit the function of p53. The homologous gene in HHV-6B is *dr6*. Since p53 is deregulated in both HHV-6A and -6B, we characterized the expression of *dr6* mRNA and the localization of the translated protein during HHV-6B infection of HCT116 cells. Expression of mRNA from *dr6* was inhibited by cycloheximide and partly by phosphonoacetic acid, a known characteristic of herpesvirus early/late genes. DR6 could be detected as a nuclear protein at 24 hpi and accumulated to high levels at 48 and 72 hpi. DR6 located in dots resembling viral replication compartments. Furthermore, a novel interaction between DR6 and the viral DNA processivity factor, p41, could be detected by confocal microscopy and by co-immunoprecipitation analysis. In contrast, DR6 and p53 were found at distinct subcellular locations. Together, our data imply a novel function of DR6 during HHV-6B replication.

## Introduction

Human herpesvirus (HHV)-6 comprises two genetically closely related variants named A and B. Despite their relatedness, HHV-6A and -6B display distinct epidemiologic and biologic characteristics, including their disease spectrum, suggesting that they are distinct species [1]. At the age of two, most individuals in the western world have been infected with HHV-6B [2], [3], which causes the childhood disease exanthem subitum [4]. The epidemiology of HHV-6A is less well known. Whereas HHV-6A is believed to be more neurotropic [5], [6], HHV-6B is frequently associated with salivary glands and may be the only HHV-6 variant associated with saliva [7]. The molecular basis for the epidemiological and biological differences between HHV-6A and -6B is poorly understood. Although both viruses have 9 variant-specific open reading frames (ORFs) [8], [9], it remains to be demonstrated whether or not they encode functional proteins.

Previously, we and others have reported that regulation of p53 is disturbed in HHV-6A and -6B-infected cells [10], [11], [12]. Major cellular pathways, including apoptosis and cell cycle arrest, are regulated by p53, and consequently, mutations in p53 are a frequent finding in many forms of cancer [13]. The protein product of *orf-1* (here termed ORF-1(6A)) identified in the *Sal*I-L fragment of HHV-6A (U1102) [14] has been reported to bind p53 [15]. Using various truncated constructs of p53, ORF-1(6A) binding was mapped to a region within residues 28 to 187, which includes the C-terminal half of the p53 transcriptional activation domain and the N-terminal half of the DNA-binding domain. Cells expressing ORF-1(6A) were inhibited in p53-activated transcription, and ORF-1(6A) was able to transform NIH3T3 cells *in vitro* and to induce fibrosarcomas *in vivo* when injected into nude mice [15]. This viral protein was predicted to be 357 amino acids [16], and has been suggested to correspond to the protein product of *direct repeat 7* (*DR7*) [9], here designated DR7(6A), although the nucleotide sequence for *dr7(6A)* encodes a predicted protein of 363 amino acids. In addition to this unspliced product, a spliced product is predicted to originate from *dr6* in both HHV-6A (395 amino acids), here designated *dr6(6A)*, and HHV-6B (392 amino acids), designated *dr6(6B)*.

Based on the transforming and *trans-*activating abilities of ORF-1(6A), DR6(6B) has also been proposed to be a *trans-*activator. To understand further the function of DR6(6B) in HHV-6B-infected cells, we have generated a polyclonal anti-DR6 antibody. Characterization of DR6(6B) expression and localization during infection suggests novel functions for this viral protein.

## Results

### Nucleotide alignments of the HHV-6A/B genomes encoding ORF-1(6A), DR7(6A), DR6(6A), and DR6(6B)

**Figure 1:**
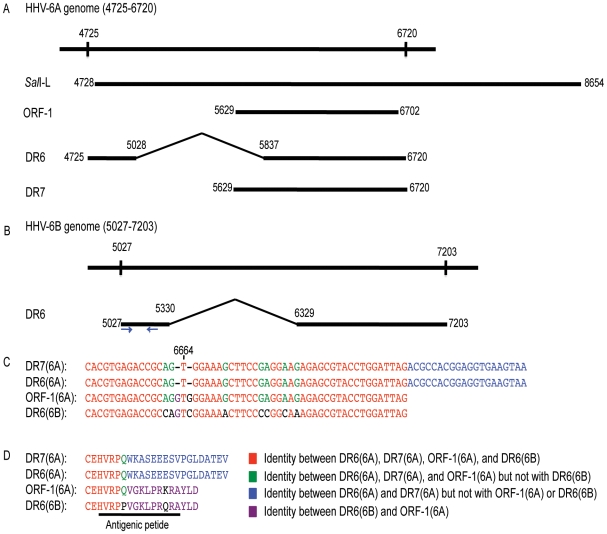
Alignments of *dr7(6A)*, *dr6(6A)*, *orf-1(6A)*, and *dr6(6B)*. (A) Schematic alignment of the *dr6* gene from HHV-6A (NC_001664, nt 4725-6720) with the *Sal*I-L fragment (X73675), the *orf-1* from the *Sal*I-L fragment [14], the *dr6(6A)* and *dr7(6A)* open reading frame [9]. (B) Schematic alignment of *dr6* gene of HHV-6B (NC_000898, nt 5027-7203). Arrows indicate the primers used for amplification by real-time PCR. (C) Alignment of the 3′-end of *dr7(6A)*, *dr6(6A)*, *orf-1(6A)*, and *dr6(6B)*. Nt 6664 indicates the position where a frameshift takes place in *dr6(6A)* and *dr7(6A)*. (D) Alignment of the C-terminal tail of DR7(6A), DR6(6A), ORF-1, and DR6(6B). The amino acid sequence for the antigenic peptide is indicated.

According to the HHV-6A nucleotide sequence (NC_001664), DR6(6A) and DR7(6A) is encoded from a region between nt 4725 and 6720 (Figure 1A). The *SalI*-L fragment (nt 4728 to 8654) includes this region with the exception of the ATG that is necessary for translation of the *dr6(6A)* coding sequence (Figure 1A). Therefore, the *orf-1(6A)* may encode DR7(6A), although it is not known whether this protein product is generated during HHV-6A infection.

In the HHV-6B genome (NC_000898), the nt 5027 – 5330 is joined to 6329 – 7203 to generate the coding sequence for DR6(6B) (Figure 1B). Similarity between *orf-1(6A)*, *dr6(6A)*, and *dr7(6A)* was expected, but surprisingly the 3′-end of the *orf-1(6A)* is almost identical to *dr6(6B)*, but not to *dr6(6A) or dr7(6A)*. Inspection of the sequences reveal that *dr6(6A)* and consequently *dr7(6A)* have a sequence that reads AGTGG as opposed to AGGTGGG in *orf-1(6A)* and CAGTCGG *dr6(6B)* (Figure 1C). Thus, the lack of a G before and after T alters the reading frame and results in the prediction of a different protein (Figure 1C and 1D). Insertion of G's in position 6664 and 6666 of *dr6(6A)* and *dr7(6A)* would maintain a reading frame comparable to *orf-1(6A)* and *dr6(6B)* and introduce an earlier stop codon. This could explain the inconsistency in length of the predicted proteins from *orf-1(6A)* (357 amino acids) and *dr7(6A)* (363 amino acids). Moreover, this insertion of G's would result in nearly identical coding sequences for *dr6(6A)* and *dr6(6B).* We propose that the multiple G's in the sequence AGGTGGG could have led to a misreading of the sequence (AGTGG) for *dr6(6A)* and *dr7(6A)*, and suggest therefore that DR6(6A) and DR6(6B) are more similar than initially predicted and may serve the same functions.

### Expression of *dr6(6B)* mRNA and protein in HHV-6B-infected cells

**Figure 2:**
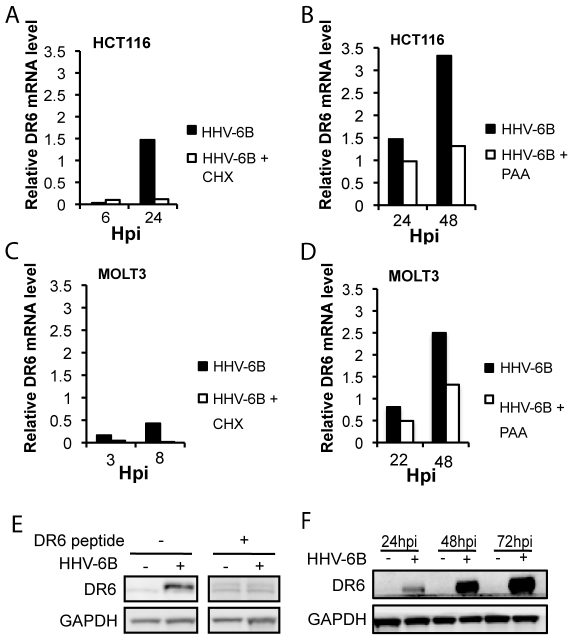
Expression of *dr6* mRNA and protein in HHV-6B-infected cells. (A–D) Relative expression of *dr6* mRNA using *tbp* mRNA as reference. HCT116 (A and B) or MOLT3 (C and D) cells were incubated in the presence or absence of CHX (A and C) or PAA (B and D) and lysed at the indicated timepoint followed by mRNA extraction. (E) Anti-DR6 polyclonal antibodies identified a 44 kDa band by Western blotting, which was blocked by the addition of a DR6(6B) peptide. The peptide sequence was as shown in Figure 1D. (F) Western blotting of HCT116 cell extract at 24, 48, and 72 hpi. Expression of DR6(6B) and GAPDH is shown. A representative of 3 experiments is shown.

To examine whether *dr6* mRNA was expressed during HHV-6B infection, we used real-time PCR. Expression of *dr6(6B)* mRNA was significant at 24 hours post infection (hpi) in both HCT116 and MOLT3 cells (Figure 2A–D). The presence of inhibitors of early (cycloheximide, CHX) and late (phosphonoacetic acid, PAA) genes, indicated that the *dr6(6B)* gene followed an expression pattern characteristic of an early/late gene, as it was inhibited by the presence of CHX (Figure 2A and 2C), and the presence of PAA had a moderate inhibitory effect (Figure 2B and 2D). The kinetics of expression was similar in HCT116 and MOLT3 cells.

To demonstrate that the presence of *dr6(6B)* mRNA resulted in translation to a viral protein, a polyclonal rabbit anti-DR6 antibody against the C-terminal fragment of DR6(6B) was used (Figure 1D). In HHV-6B-infected cells, this antibody recognized a protein of an apparent MW of 44 kDa that was not recognized in the presence of the blocking peptide against which the antibody was raised (Figure 2E). Using this antibody, the expression of DR6(6B) in HHV-6B-infected HCT116 cells was clearly detectable at 24 hpi, and the expression increased dramatically at 48 and 72 hpi (Figure 2F). This indicated that high levels of DR6(6B) were expressed during HHV-6B infection.

### Different localization of DR6 and p53 in HHV-6B-infected cells

**Figure 3:**
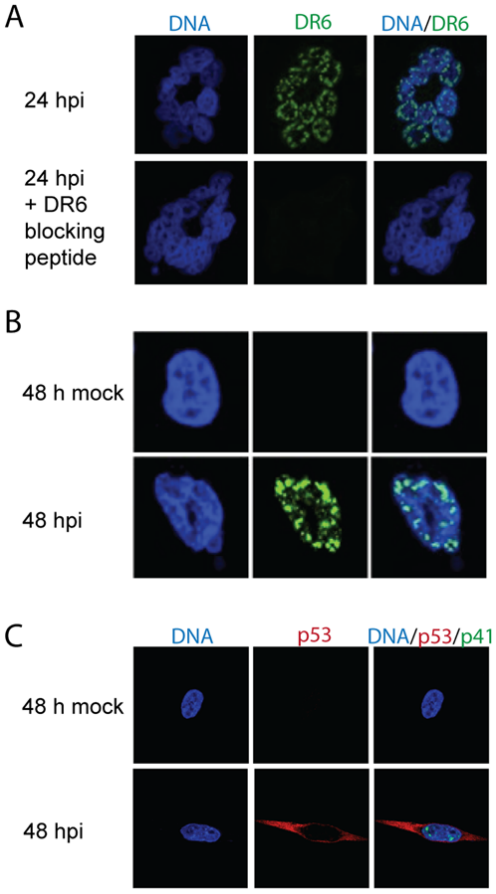
Separate localization of DR6(6B) and p53 in HHV-6B-infected cells. (A) Confocal laser scanning microscopy of DR6(6B) (green) at 24 hpi in the presence or absence of a DR6(6B) blocking peptide. The peptide sequence is shown in Figure 1D. DNA was stained with DAPI (blue). Multiple cells forming a characteristic ring structure are shown. (B) Distribution of DR6(6B) (green) at 48 hpi. A representative cell is shown. (C) Distribution of p53 (red) and p41 (green) at 48 hpi. The size of the cells in (A), (B) and (C) are similar, but (B) is shown at 3x higher magnification than are (A) and (C). A representative of three experiments is shown.

In HHV-6A-infected cells, DR7(6A) has been proposed to bind p53 and inhibit its transcriptional activity. Since p53 transcriptional activity also appears to be inhibited in HHV-6B-infected cells, we examined whether DR6(6B) co-localized with p53 in HHV-6B-infected HCT116 cells. To verify that our anti-DR6 antibody did not give rise to non-specific signals on confocal microscopy, we first examined the expression of DR6(6B) at 24 hpi in the presence or absence of the blocking peptide against which the antibody was raised. This indicated that our anti-DR6 antibody did not bind non-specifically through sites other than the antigen-specific site (Figure 3A). Confocal microscopy identified DR6(6B) at 24 and 48 hpi (Figure 3A and B), consistent with the Western blotting analysis (Figure 2F). Whereas DR6(6B) was located in the nucleus, the distribution of p53 was almost exclusively cytoplasmic in HCT116 cells that were HHV-6B infected, as judged by expression of p41 (Figure 3C). As expected, uninfected HCT116 cells expressed undetectable levels of p53. This demonstrated that DR6(6B) and p53 localize to different cellular compartments during HHV-6B infection.

### Nuclear distribution of DR6 in a pattern similar to the distribution of TBP

**Figure 4:**
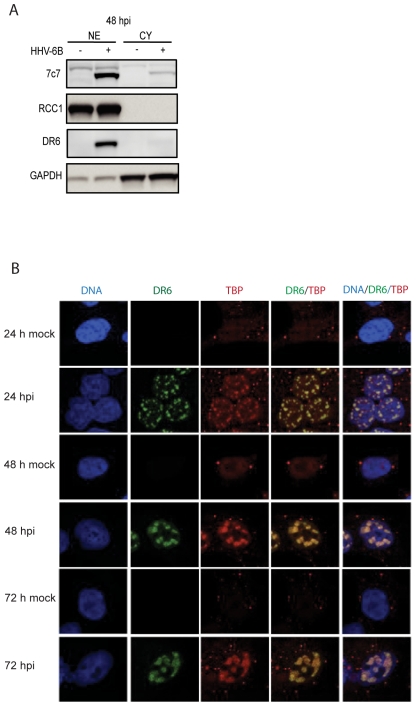
Nuclear distribution of DR6(6B) in a pattern similar to TBP. (A) Separation of HCT116 lysate at 48 hpi into nuclear (NE) and cytoplasmic (CY) fractions. Western blots were probed with anti-DR6, anti-7C7 to indicate HHV-6B infection, anti-RCC1 as a nuclear protein control, and anti-GAPDH as a cytoplasmic control. (B) Confocal laser scanning microscopy of localization of DR6 and TBP at 24, 48, and 72 hpi. DNA was stained with DAPI. Columns with DR6/TBP and DNA/DR6/TBP indicate an overlay of the individual stainings. A representative of three experiments is shown.

The distribution of DR6(6B) observed by confocal microscopy suggested that it was almost exclusively nuclear. To further demonstrate this, we separated nuclear and cytoplasmic fractions from HHV-6B-infected HCT116 cells at 48 hpi. This indicated that the vast majority of DR6(6B) was found in the nuclear fraction with barely detectable levels in the cytoplasmic fraction (Figure 4A). Controls indicated that the cytoplasmic fractions were pure, since we were unable to detect the nuclear protein RCC1, whereas there was some cytoplasmic contamination in the nuclear fractions, as seen by the presence of GAPDH. This does not affect our conclusion that DR6(6B) was almost exclusively found in the nucleus during HHV-6B infection of HCT116 cells at 48 hpi.

To determine the distribution of DR6(6B) during the infection, HHV-6B-infected HCT116 cells were examined at 24, 48, and 72 hpi. At the earliest timepoint examined, DR6(6B) appeared in the nucleus and increased its intensity significantly at 48 and 72 hpi. The staining pattern were reminiscent of a pattern seen in HSV-1-infected cells by TATA binding protein (TBP) [17], a transcription factor that is part of the RNA polymerase II preinitiation complex. TATA box motifs may be present in promoters for a number of viral genes. There was a high degree of co-localization between DR6(6B) and TBP (Figure 4B), suggesting that these molecules were functioning in the same nuclear compartment. When the cells were infected by HHV-6B, an increased staining of TBP was detected. The anti-TBP antibody detects an epitope that is exposed when TBP binds DNA. Since TBP is not upregulated by HHV-6B infection (data not shown), the most likely explanation of the results is an increased binding of TBP to DNA during HHV-6B infection.

### Association of DR6 with the p41 viral DNA processivity factor

**Figure 5:**
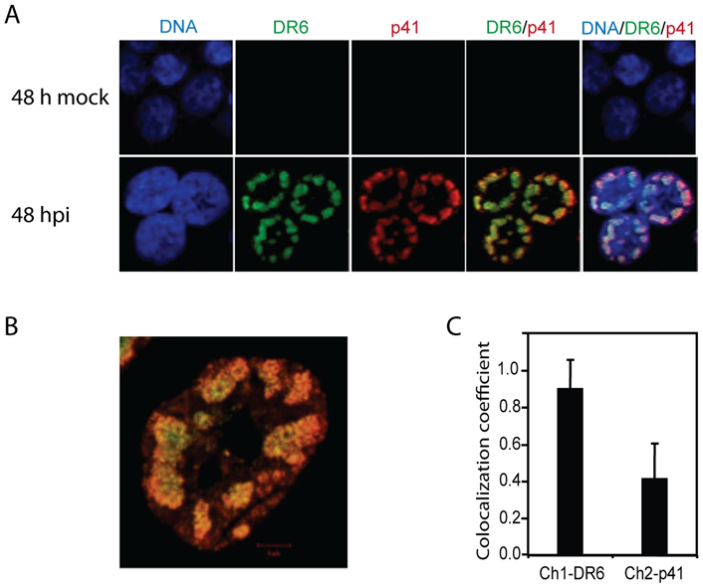
Association of DR6(6B) and the p41 viral DNA processivity factor. (A) Confocal laser scanning microscopy of DR6(6B) (green) and p41 (red) of HCT116 cells at 48 hpi. DNA was stained with DAPI (blue). Columns with DR6/p41 and DNA/DR6/p41 indicate an overlay of the individual stainings. (B) High magnification of DR6/p41 overlay. (C) Co-localization coefficient computed by the LSM710 software. Ch1-DR6 indicates the fraction of DR6(6B) that co-localized with p41. Ch2-p41 indicates the fraction of p41 that co-localized with DR6(6B). Values are the average of measurements on 14 cells with an indication of standard deviation on top of the bars. A representative of three experiments is shown.

The distribution pattern of DR6(6B) also resembles replication compartments of viral DNA, as has been described for HSV-1 [18]. To pursue a potential role of DR6(6B) in viral DNA replication, we examined the potential co-localization between DR6(6B) and the viral DNA polymerase processivity factor p41. Overall, DR6(6B) and p41 localized to the same compartments within the nucleus of HHV-6B-infected HCT116 cells (Figure 5A). Overlay analyses indicated that the majority of DR6(6B) co-localized with p41, although p41 was more dominant at the border of the nucleus. (Figures 5A and B). Software to calculate the co-localization indicated that almost 90% of DR6(6B) co-localized with p41, whereas only 40% of p41 co-localized with DR6(6B) at 48 hpi (Figure 5C).

### Complex formation of DR6(6B) and p41 during HHV-6B infection

**Figure 6:**
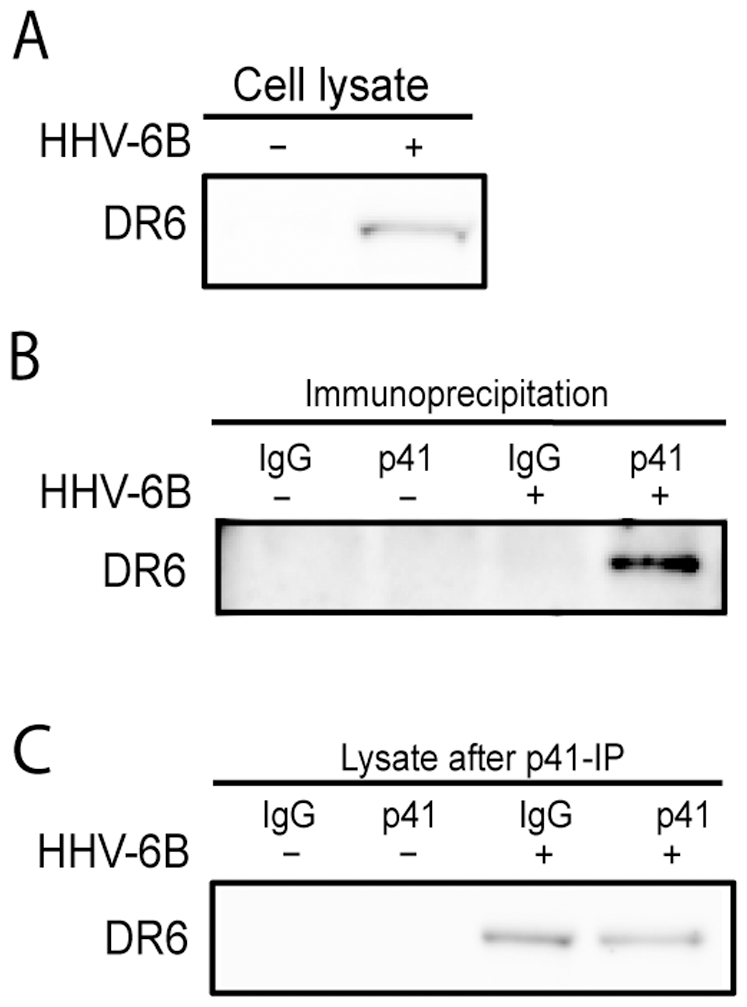
Complex formation of DR6(6B) and p41 during HHV-6B infection. (A) Western blotting of input lysate before immunoprecipitation. Probed with anti-DR6. (B) Western blotting of p41 and control IgG immunoprecipitation with or without HHV-6B-infection at 48 hpi. Probed with anti-DR6. (C) Western blotting of lysates after one round of p41 and IgG immunoprecipitation. Blots were probed with anti-DR6. A representative of two experiments is shown.

Co-localization by confocal microscopy suggests that DR6(6B) and p41 may have a functional relationship. To examine whether these proteins physically associated in the same complex, immunoprecipitates of p41 were probed for the presence of DR6(6B). This demonstrated that DR6(6B) in the input lysate (Figure 6A) co-immunoprecipitated with anti-p41, but not with irrelevant IgG (Figure 6B). The post-immunoprecipitated lysate indicated that a fraction of the DR6 was removed by anti-p41 when compared with irrelevant IgG (Figure 6C). Together, these data indicated that DR6(6B) and p41 form a complex during infection, either directly or indirectly.

## Discussion

A major protein in controlling the antiviral response is p53, the function of which is inhibited during HHV-6B infection. Early experiments reported that the protein product from ORF-1 of the *Sal*I-L fragment of HHV-6A strain U1102 might bind to p53, interfere with its transcriptional activity, leading to fibrosarcomas in nude mice and transformation in NIH3T3 cell lines [15]. Consistent with the alignment shown in Figure 1, *orf-1(6A)* may encode a protein from *dr7(6A)*, since the *Sal*I cleavage removes the ATG from the *dr6(6A)* open reading frame. However, it has never been shown that *dr7(6A)* encodes a protein during infections.

Based on these early studies, we speculated that DR6(6B) might in part explain the altered p53 regulation in HHV-6B-infected cells. To address this, we characterized the localization of DR6(6B) during HHV-6B infection using the anti-DR6 antibody. Whereas the vast majority of p53 was located in the cytoplasm during infection in HCT116 cells, DR6(6B) was almost exclusively detected in the nucleus. Although remarkable, this dichotomy in localization would not definitively rule out an association between DR6(6B) and p53 in the nucleus. Nonetheless, based on the distribution of p53 and DR6(6B) within the cellular compartments, our data do not support the hypothesis that DR6(6B) acts as an inhibitor of p53 during HHV-6B infection.

The above conclusion appears to be inconsistent with the results from Kashanchi *et al*. [15]. The reason for this is not clear, but we speculate that during ORF-1 overexpression, p53 and ORF-1 may both localize to the nucleus. In contrast, during HHV-6B infections, p53 is deregulated and the vast majority is located in the cytoplasm, whereas DR6(6B) is found in the nucleus. Moreover, the protein product of *orf-1(6A)* must be different from the product of *dr6(6B)*, since the ATG for *dr6(6A)* is cleaved by the *SalI* enzyme and an alternative ATG is used for *dr7(6A)*.

Confocal microscopy identified DR6(6B) in major nuclear dots resembling replication compartments. These structures also contained TBP and p41 indicative of viral transcription and replication. Importantly, p41 immunoprecipitation was able to co-precipitate DR6(6B). This suggests that p41 and DR6(6B) are present within the same complex involved in viral DNA replication. We do not know whether p41 and DR6(6B) interacts physically, since DR6(6B) may be co-precipitated via other proteins in the complex. Cloning and expression of these proteins should illuminate potential interactions between them in greater detail. Our confocal microscopy studies have already demonstrated a high degree of co-localization, although this was not complete. At least 10% of DR6(6B) was located in the dots in the absence of p41 and almost 60% of p41 was located in the dots in the absence of DR6(6B). It is unclear whether masking of antibody epitopes through protein-protein or protein-DNA interactions may interfere with the identification of the proteins and thus with these estimates, but we cannot exclude the possibility that DR6(6B) is associated with another protein in the complex.

DR6(6B) displayed a rapid nuclear translocation, although its sequence has no known nuclear localization motif. This suggests that DR6(6B) may use a novel motif or that it may be transported to the nucleus by another protein, potentially a protein that directs DR6(6B) to the replication compartments. After 48 to 72 hpi the level of DR6(6B) increased steeply. Whether other viral proteins produced before 24 hpi is important for the significant increase in DR6(6B) levels or whether DR6(6B) via a positive feed-back loop may transactivate itself remains to be defined. The latter possibility would be consistent with a transactivating function of DR6(6B). Either way it suggests the possibility that DR6(6B) is an important protein for the further progression of the infection and is potentially necessary for viral replication. We have attempted to knock-down DR6(6B) protein levels using siRNA, but were unsuccessful in eliminating DR6(6B) to an undetectable level judged by confocal microscopy, even when specific siRNA was present before the infection. To define definitively the role of DR6(6B) during HHV-6B replication may therefore require a *dr6(6B)* mutant virus, which is not available at the moment. In addition, although viral DNA appears to be produced in HCT116 cells (data not shown), this cell line is not thought to be productively infected, although this has not been examined in detail.

In conclusion, we have characterized the expression pattern of *dr6* mRNA and the localization of the translated protein during HHV-6B infection of HCT116 cells. We have further identified a novel interaction between DR6(6B) and the viral DNA processivity factor, p41, by confocal microscopy and by co-immunoprecipitation analysis. In contrast, DR6 and p53 were found at distinct subcellular locations. In addition to the previously reported transactivating function on heterologous promoters, these studies imply a novel function of DR6(6B) during viral DNA replication.

## Materials and Methods

### Cell lines and virus stock

The human epithelial colon carcinoma cell line HCT116 (a gift from B. Vogelstein and K.W. Kinzler, Johns Hopkins University School of Medicine, Baltimore, MD, USA), was grown in McCoy's medium (Gibco, California, USA), supplemented with 10% inactivated fetal calf serum (Sigma-Aldrich, Saint Louis, USA), glutamine (0.2 g/L), HEPES (10 mM), penicillin (0.2 g/L), streptomycin (0.2 g/L) (all from the Substrate Department, Bartholin Building, Aarhus University, Denmark).

The immature T-cell line MOLT3 was grown in Isocoves Modified Dulbecco Medium (IMDM) supplemented with 10% inactivated fetal calf serum (Sigma), glutamine (0.2 g/L), HEPES (10 mM), penicillin (0.2 g/L), streptomycin (0.2 g/L) (all from the Substrate Department).

HHV-6B, strain PL1 (generated by Dr. P. Lusso, Milan, Italy), was propagated in MOLT3 cells in IMDM supplemented with 10% inactivated fetal calf serum (Sigma), 0.5% nystatin (Gibco), glutamine (0.2 g/L), HEPES (10 mM), penicillin (0.2 g/L), streptomycin (0.2 g/L) (all from the Substrate Department). Virus was collected from supernatants of HHV-6B infected MOLT3 cells by centrifugation at 3,200 xg for 1 hour to remove cell debris, followed by a centrifugation at 25,000 xg at 4° C for 3 hours to pellet virus particles. The virus particles were resuspended in IMDM and the viral titer was determined in a Reed and Munch assay to be 75 TCID_50_ [19]. Prior to each experiment including HHV-6B infection, the virus stock was diluted 1∶10.

### Alignments

Using the BLOSUM62 alignment algorithm from CLCbio workbench (Aarhus, Denmark) the nucleotide sequences from *SalI*-L fragment of HHV-6A (X73675) [14], *orf-1(6A)* from *SalI*-L [14], *dr6(6A)* and *dr7(6A)* (NC_001664) [9] were aligned against *dr6(6B)* from HHV-6B (NC_000898)[8]. Furthermore, the predicted protein sequences from DR7(6A), DR6(6A), ORF-1(6A), and DR6(6B) were aligned.

### RNA extraction

HCT116 cells (5×10^5^) were infected with HHV-6B diluted 1∶15 in McCoy's medium. RNA was isolated at 6, 24 and 48 hpi. An equal amount of infected cells were treated with either 50 mg/ml CHX (Sigma-Aldrich, Vallensbæk Strand, Denmark) or 200 µg/ml PAA (Sigma-Aldrich) at 6, 24, and 48 hpi. For RNA isolation, the cells were washed in phosphate-buffered saline (PBS) and total RNA was isolated at RT using a High Pure RNA Isolation kit (Roche Diagnostics Scandinavia AB, Hvidovre, Denmark) as described previously [20]. RNA was eluted in nuclease-free, sterile, double-distilled H_2_O in a total volume of 50 µl. Total RNA concentrations were determined by absorbance measurements using a NanoDrop 1000 instrument (Thermo Scientific, Wilmington, DE, USA).

### cDNA synthesis and real-time PCR

Purified RNA was briefly incubated in gDNA Wipeout Buffer (Quantitect Reverse Transcriptase kit, Qiagen, Copenhagen, Denmark) at 42°C for 2 minutes to remove contaminating genomic DNA. Subsequently, cDNA was synthesized from 0.5–1 µg RNA using Quantitect Reverse Transcriptase kit. Amplification of cDNA was performed in duplicate by real-time PCR on a LightCycler instrument (Roche Diagnostics) in the presence of 2 µl cDNA, 3.5 mM MgCl_2_, 0.5 µM primers and 5 µl QuantiTect SYBR Green (Qiagen) in a final volume of 10 µl. Each run included a positive control sample for amplification of the *tbp* gene and negative controls for each primer pair in the presence or absence of cDNA. The primers used for amplification of *dr6* and tbp were: 5′-ATGACAACGCGACACACG-3′ and 5′-ATCCGGATGGAGTTCTAGGC-3′ (*dr6*) and: 5′-GCGGTTTGCTGCGGTAATCAT-3′ and 5′-GACTGTTCTTCACTCTTGGCTCCTGT-3′ (*tbp*). The optimal condition was determined to be an initial denaturing step at 95°C for 10 min, followed by 45 cycles of 95°C for 15 s, 62°C for 20 s and 72°C for 15 s. Melting curves were performed with one cycle at 95°C for 0 s, 72°C for 15 s, and 99°C for 0 s. The relative amount of mRNA for DR6 was determined by the real-time PCR crossing point (Cp) and normalized against the crossing point of *tbp* amplification using the formula: 2^Cp(*tbp*) – Cp(*DR6(6B)*)^.

### Generation of anti-DR6 polyclonal antibodies

Custom-made rabbit anti-DR6 polyclonal antibodies were generated by Gencript, Piscataway, NJ, USA. In brief, rabbits were immunized with a 14-amino acid peptide from the DR6 C-terminal (Figure 1D) in order to generate a rabbit polyclonal anti-DR6 antibody. The peptide was conjugated to a keyhole limpet hemocyanin (KLH) carrier. Conjugated peptide (0.5 mg) and complete Freund's adjuvant was subcutaneously administered at day 1, and the rabbits were reimmunized with 0.5 mg KLH-conjugated peptide and incomplete Freund's adjuvant at day 14, 35, and 56 followed by a production bleed at day 70. The anti-DR6 antibody was affinity purified and the purity was confirmed by HPLC analysis. The specificity of the anti-DR6 antibody was tested using an ELISA assay.

### Cell lysis and Western blotting

HCT116 cells (2×10^6^) were either mock-treated or infected with HHV-6B and lysed at 24, 48 or 72 hpi. Cells were lysed for 30 min in 1× lysis buffer (Cell Signaling, Technology Inc., Beverly, MA, USA) supplemented with 1 mM phenylmethanesulphonylfluoride (PMSF), 5 mM sodium fluoride (NaF) and Complete Mini Protease Inhibitor (Roche Diagnostics) according to the manufacturers recommendations. Lysates were centrifuged at 4200 *×g* for 5 min, followed by a 10 min centrifugation at 20.000 *×g* to remove cell debris completely. Nuclear and cytoplasmic extracts were prepared from T cells (nuclear extract kit; Active Motif, Rixensart, Belgium) with and without HHV-6B infection at 48 hpi as previously described [12]. Protein concentrations of the lysates were measured by the Bradford method and 30 µg protein of each lysate were separated on an XT Criterion 10% gel (Bio-Rad, Copenhagen, Denmark) with 1 × XT MOPS running buffer (Bio-Rad) for 1 hour at 175 V (constant) and subsequently electro-transferred to a nitrocellulose membrane at 300 mA (constant) for 1.5 hours.

The following antibodies were used for Western blotting: Rabbit anti-DR6 antibody (1∶1000); rabbit anti-GAPDH antibody (1∶2000) (Santa Cruz Biotechnology, Santa Cruz, CA, USA); mouse anti-p41 antibody (1∶500) (Advanced Biotechnologies, Tebu-Bio, Columbia, USA); mouse anti-7c7 antibody (1∶600) (Argene Biosoft, Verniolle, France); goat anti-RCC1 antibody (1∶500) (Santa Cruz Biotechnology); horseradish peroxidase-conjugated polyclonal swine anti-rabbit antibody (P0217); horseradish peroxidase-conjugated polyclonal rabbit anti-mouse antibody (P0260); horseradish peroxidase-conjugated polyclonal rabbit anti-goat (P0449) (all from Dako, Glostrup, Denmark). All of the peroxidase-conjugated antibodies were used at 1∶2000 dilution in 5% skimmed milk in TBS with 0.1% Tween 20 (Sigma-Aldrich). Membranes were developed with Chemiluminescence pico or femto (Pierce, Thermo Scientific, Slangerup, Denmark) and Image Reader Las-3000 (Science Imaging Scandinavia AB, Sweden).

### Confocal laser scanning microscopy

HCT116 cells were transferred to poly-L-lysine-coated slides and incubated for 24 h. Cells were mock or HHV-6B infected and incubated for additional 24, 48, or 72 h, followed by fixation in 4% formalin/PBS (pH 7.5). Cells were washed twice in PBS, permeabilized in 0.2% Triton X-100 (Sigma-Aldrich)/PBS and blocked in 5% BSA (Sigma-Aldrich)/PBS. DR6 was visualized using rabbit anti-DR6 antibody (1∶200) and p53 was visualized with a rabbit anti-p53 antibody (1∶200) (Santa Cruz Biotecnologies). TBP was visualized using mouse anti-TBP antibody (1∶200) (Abcam, Cambridge, UK) and p41 was visualized using mouse anti-p41 antibody (1∶500). The secondary antibodies were a goat anti-rabbit F(ab')_2_ antibody conjugated with Alexa Fluor 488 (Invitrogen) (1∶400) and a goat anti-mouse F(ab')_2_ antibody conjugated with Alexa Fluor 546 (Invitrogen) (1∶400). Nuclear staining was performed with the DNA dye 4′,6-diamidino-2-phenylindole dihydrochloride (DAPI), (Sigma-Aldrich). Imaging was done by using a 488 nm line of a multiline argon laser, the 543 nm line of the green helium–neon laser, and the 633 nm line of the helium–neon laser on a confocal laser scanning microscope (LSM710, Zeiss, Jena, Germany) with a x63 oil-immersion objective with a numerical aperture of 1.4.

### Co-immunoprecipitation

HCT116 cells (2×10^6^) were either mock-treated or infected with HHV-6B and incubated for 72 h before cell lysis. Antibodies were coupled to protein A Dynabeads (Invitrogen) using 5 µg mouse anti-p41 antibody or control mouse-IgG_2a_ (Sigma-Aldrich) by rotating the mixture for 10 min at room temperature. Beads were washed twice in PBS and the antibody-bead binding was crosslinked with 5 mM subericacid bis (3-sulfo-N-hydroxysuccinimide ester) sodium salt (BS^3^) (Sigma Aldrich) for 30 min at RT, after which the crosslinking was quenched using 1 M TrisHCl (pH 7,5). Finally the antibody-conjugated beads were incubated with 400 µg of protein lysate for 10 min at 4°C, followed by 3 washing steps in PBS supplemented with 0.1% Tween 20 (Sigma-Aldrich). Bound protein was eluted with 5 volumes of 4x sample buffer (Bio-Rad) and 1 volume of reducing agent (Bio-Rad).
